# Integration of Consumer-Based Activity Monitors into Clinical Practice for Children with Type 1 Diabetes: A Feasibility Study

**DOI:** 10.3390/ijerph182010611

**Published:** 2021-10-10

**Authors:** Jason R. Jaggers, Timothy McKay, Kristi M. King, Bradly J. Thrasher, Kupper A. Wintergerst

**Affiliations:** 1 Wendy Novak Diabetes Center, Department of Pediatrics, Division of Endocrinology, School of Medicine, University of Louisville, Norton Children’s Hospital, Louisville, KY 40202, USA; timothy.mckay@louisville.edu (T.M.); kristi.king@louisville.edu (K.M.K.); bradly.thrasher@louisville.edu (B.J.T.); kupper.wintergerst@louisville.edu (K.A.W.); 2 Department of Health and Sport Sciences, University of Louisville, Louisville, KY 40292, USA

**Keywords:** physical activity, pediatric, clinical exercise, accelerometer, diabetes

## Abstract

Current technology commonly utilized in diabetes care includes continuous glucose monitors (CGMs) and insulin pumps. One often overlooked critical component to the human glucose response is daily physical activity habits. Consumer-based activity monitors may be a valid way for clinics to collect physical activity data, but whether or not children with type 1 diabetes (T1D) would wear them or use the associated mobile application is unknown. Therefore, the purpose of this study was to test the feasibility of implementing a consumer-based accelerometer directly into ongoing care for adolescents managing T1D. Methods: Adolescents with T1D were invited to participate in this study and instructed to wear a mobile physical activity monitor while also completing a diet log for a minimum of 3 days. Clinical compliance was defined as the number of participants who were compliant with all measures while also having adequate glucose recordings using either a CGM, insulin pump, or on the diet log. Feasibility was defined as >50% of the total sample reaching clinical compliance. Results: A total of 57 children and teenagers between the ages of 7 and 19 agreed to participate in this study and were included in the final analysis. Chi-square results indicated significant compliance for activity tracking (*p* < 0.001), diet logs (*p* = 0.04), and overall clinical compliance (*p* = 0.04). Conclusion: More than half the children in this study were compliant for both activity monitoring and diet logs. This indicates that it is feasible for children with T1D to wear a consumer-based activity monitor while also recording their diet for a minimum of three days.

## 1. Introduction

With overwhelming evidence supporting the value of daily physical activity [1], healthcare providers educate their patients that staying physically active improves cardiovascular health, increases insulin sensitivity, and improves mental health among many other benefits. There have been calls to action for healthcare professionals to include measures of physical activity and exercise in a standard assessment during routine clinical visits for use as a vital sign that is kept as a health indicator in their medical record, similar to blood pressure and weight [1,2,3,4,5]. Unfortunately, obtaining verifiably accurate activity data from patients has been difficult, with most providers relying on self-reported times and individual perceptions of activity degree (i.e., moderate vs. vigorous) [3,5]. Whether or not it would be feasible to incorporate data from a physical activity monitor for patient management into standard practice is unknown. Utilizing this technology would provide healthcare providers with accurate activity data and improve specific time and activity level counseling. Furthermore, to continue to explore the range of activity associated with health in children, studies involving accelerometers could provide valid and reliable measurements for household and sedentary behaviors that often go under-reported when communicating with healthcare providers [6]. One such clinical population that could benefit from physical activity monitoring is type 1 diabetes (T1D). With the assistance of an exercise physiologist, physicians can incorporate individualized recommendations for increasing physical activity and/or exercise prescriptions into their clinical practices. This ensures the patient’s exercise regimen is both safe and effective [2,7].

With all children encouraged to accumulate at least 60 min of play for known health benefits [8], providers caring for children with T1D must determine how best to advise so that participation can be free from fear of diabetes-related complications. This is particularly challenging because children with T1D often have frequent changes in carbohydrate needs and insulin requirements as they grow. This is especially true during periods of more rapid growth and development, as well as acutely with changes in physical activity. During moderate-to-vigorous physical activity, there are significant changes in blood glucose concentration [9,10,11], and poor glycemic control can lead to the impairment of physical growth and a delay in pubertal development [12]. Utilizing activity monitoring technology could help medical professionals involved in T1D care make more accurate adjustments to insulin therapy and diet recommendations. Children with T1D that maintain good glycemic control do not display signs of impaired muscle function, while children with poor glycemic control can have altered aerobic muscle capacity [11,13]. Hypoglycemia, during or within hours following large bouts of increased activity or planned exercise, can interfere with the activity or even cause potential harm [14]. In fact, many children with T1D are inactive due to the fear of, and possible prior experiences with, hypoglycemia or hyperglycemia [15,16]. 

The American Diabetes Association’s position statement “Type 1 Diabetes through the life span” recommends physical activity and exercise [17,18]. This statement provides examples of reviews that have been published regarding consensus on exercise management for individuals with T1D who exercise regularly, including glucose targets for safe and effective exercise, and nutritional and insulin dose adjustments to protect against exercise-related glucose excursions [7,19]. Unfortunately, these recommendations are relatively general and based primarily on research studies with adults managing type 2 diabetes. Individuals managing T1D need recommendations that are tailored to their individual needs. Providers have been increasingly turning to diabetes technology to better serve their patients, and the use of activity monitoring technology would be another valuable step. 

The advancement of diabetes technology, including continuous glucose monitoring (CGM) devices, continuous subcutaneous insulin pumps, and even hybrid closed-loop systems, has demonstrated direct value from increasing data input from the patient. Direct patient activity data are not yet a factor in these technologies yet clearly influence glucose change. This area of research is steadily growing, and, similar to study comparisons between glucose monitoring devices, activity monitoring devices are also being evaluated. In 2018, the American Heart Association investigated the validity and feasibility of wearable activity monitoring devices for patients and healthcare data integration. Accelerometers were scored based on variables regarding the validity, test–retest reliability, and even clinical feasibility among other criteria [3]. With consumer-based activity monitors only improving, it seems ideal to use this form of technology for T1D management. However, wearing or even carrying too many devices (i.e., continuous glucose monitors and insulin pumps) has been reported as a barrier for the adoption of current diabetes technology, which could limit its use. 

The relationship between physical activity and glycemic control is complex because the acute and prolonged glucose response experienced is highly variable among patients managing T1D and dependent on both the intensity and duration of physical activity [7,9,13,19]. For example, prolonged aerobic activity causes blood glucose values to decline, whereas brief, intense anaerobic exercises causes blood glucose values to rise. This complexity has become clearer from past studies that utilized accelerometers in this population showing risk of nocturnal and next day hypoglycemia that was identified in children hours after engaging in physical activity durations longer than one hour [9,18,19]. Further risk has been identified when engaging in physical activity during late afternoon or early evening hours, which are common times for many adolescent sporting events or competitions [7,13,19]. Having a reliable tool available, such as accelerometers, would help the patient and their diabetes care team to identify these short and/or prolonged bouts more objectively and take necessary precautionary measures by providing an individualized plan of action before, during, and after said activity, thus allowing for improved glycemic control. Furthermore, to alleviate dysglycemia for those engaging in physical activity, recreationally or during school-based activities such as gym class or recess, knowing the type of activity the patient engaged in, at what intensity level, and for how long provides crucial information that allows for better adjustments to the patient’s diabetes treatment regimen. 

Another important factor to include that influences both glucose and activity is food intake. Activity and dietary intake data collected and analyzed together provide a more comprehensive view of the influences on glucose for diabetes management. Historically, certified diabetes clinical and educator specialists (CDCES) have relied primarily on written logs from patients to assess carbohydrate intake. However, more robust diet logs or even technologies such as smartphone apps could offer a more in-depth overview of food intakes such as total calories, fat, protein, and fiber intake. However, obtaining information about food intake is time consuming, which is a significant barrier for appropriate analysis of this information. With the availability of more advanced technology and having multiple tools available to record dietary intake, it is just as important to explore more advantageous ways to collect dietary information that a CDCES can analyze quickly for appropriate analysis.

Therefore, the purpose of this investigation was to test the feasibility of integrating a consumer-based physical activity monitor into an established pediatric specialty clinic to collect physical activity data for a minimum of three days. We also sought to determine the feasibility of collecting a complete diet log on the same three days in which the activity monitor was worn since carbohydrate and protein intake directly impact blood glucose, providing further insight for providers to make more informed recommendations for their patient’s diabetes management plan. We hypothesized that full compliance in wearing the activity monitor and recording dietary intake for three days as instructed would be reached by >50% of the total sample, indicating feasibility.

## 2. Materials and Methods

### 2.1. Study Design

This study is a single cohort design to assess the feasibility of using an activity monitor in combination with diet intake data collection in children with T1D. The primary aim was to determine if children with T1D would wear an activity monitor while also tracking their diet for a minimum of three days using either a smartphone app or paper log. Feasibility was defined as >50% of the total sample reaching clinical compliance, as described below.

### 2.2. Participants

Children and adolescents aged 7 to 19 with T1D receiving care at the Wendy Novak Diabetes Center at Norton Children’s and the University of Louisville were invited to participate in this study. Eligible criteria included individuals with a diagnosis of T1D and who agreed to the following: (1) Willing to wear a physical activity monitor during the study period. (2) Log diet for 3 days using either a paper log or a dietary app on their smartphone. (3) Provide daily glucose values from the same days in which diet was logged and the activity monitor was worn by allowing researchers access to their wireless CGM reports or on a paper log if no CGM is available. The study was approved by the University Institutional Review Board (Approval # 18.0713). Parental consent and child assent were obtained for all participants under 18 years of age.

### 2.3. Study Procedures

After obtaining assent and/or consent, each participant was provided a Fitbit Charge 2 physical activity monitor and the option to utilize a paper log or a mobile application to collect diet information. Clinical chart data at consent and during the study period were collected, including demographic information, HbA1c measurements on record <1 month in which activity monitor was worn, next available HbA1c measurement on record following retrieval of activity monitor, and if applicable their exported CGM, glucose meter, and insulin pump data covering the same timeframe that the activity monitor was worn.

Each participant was also provided a unique email and password combination to create an account on Fitbit.com. The account was connected to the Fitbit provided to each participant throughout the study duration. Participants were given the associated email/password so that they could log in to the Fitbit.com user account to log their diet if they did not want to turn in a paper copy and use the online dashboard and associated mobile applications. All participants were instructed to log their diet a minimum of 3 days at the same time they wore the activity monitor, insulin pump (if applicable), and CGM. Three days was chosen for clinical feasibility since it was determined by medical staff that having at least 3 days of a complete diet and activity habits to review would be adequate to make informed decisions regarding dietary recommendations and insulin adjustments to improve diabetes management.

Compliance was determined for each introduced measure including the diet log and activity monitor. In order to be compliant with the activity monitor, participants had to have a minimum of 3 days with at least 10 hours or more of wear time while awake. Dietary compliance was determined if participants recorded their diet for a minimum of the 3 days that they also wore the activity monitor. Clinical compliance was defined as the number of participants who were compliant with all measures while also having adequate glucose recordings using either a CGM, insulin pump, or on the diet log. These variables were chosen because they are collected during routine standard of care visits with their diabetes care team due to their known influence on blood glucose. 

### 2.4. Data Analysis

All data were exported into an Excel spreadsheet for analysis using SPSS. Chi-square tests were used for dichotomous variables coded according to compliant (1) or non-compliant (0) with activity monitor only, diet only, or both (clinical compliance). As previously stated, feasibility was defined as >50% of the total sample reaching clinical compliance. Since there was an uneven distribution of sample sizes between groups, a Kruskal–Wallis non-parametric test of independent samples was used to compare average daily glucose for those in compliance with those who were not. A normality test indicated HbA1c was shown to follow a normal distribution, so a paired samples *t*-test was used to test for differences in HbA1c within each group.

## 3. Results

**Figure 1:**
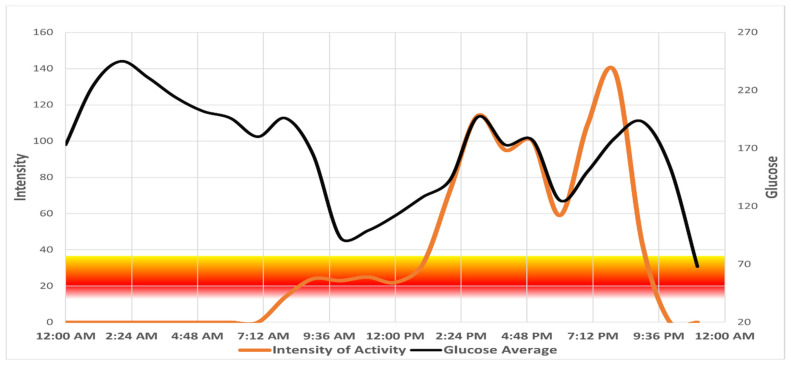
Graph of single day hourly averages of activity monitor and CGM measures. Intensity of activity values indicated on left side of *y*-axis and glucose measures obtained from CGM on right.

A total of 57 children and teenagers between the ages of 7 and 19 were enrolled in the study and included in the analysis. Participant demographics for the entire sample as well as comparisons of compliant vs. non-compliant individuals are presented in Table 1. Over the study period, 84% of participants were compliant with activity monitoring and 63% successfully recorded their diet for at least 3 days. When looking at clinical compliance, there were a total of 36 participants (63%) who completed both on the same days in which they wore their CGM, or recorded glucose numbers on the diet log (Table 2). Chi-square results indicated significant compliance for activity tracking (*p* < 0.001), diet logs (*p* = 0.04), and overall clinical compliance (*p* = 0.04). A Kruskal–Wallis non-parametric test of independent samples showed no significant difference in average daily glucose or HbA1c between groups. However, within the compliant group, a paired samples *t*-test indicated a decreasing trend that nearly reached significance (*p* = 0.09) was observed in HbA1c levels, which went from 8.19 ± 1.25% to 7.84 ± 1.13% after receiving a summary of results in the form of daily activity and glucose graphs. Figure 1 shows a single-day graph created using Excel of a compliant participant as an example, with hourly intensity values (orange line) indicated on the left side of the y-axis and glucose measures obtained from the CGM (black line) on the right side of the y-axis.

## 4. Discussion

This study sought to test the feasibility of incorporating activity monitors and diet logs as a form of mobile data collection in an established pediatric diabetes specialty clinic. Our intent was not to intervene with current medical recommendations, but simply to establish whether or not a convenience sample of a clinical population would wear an activity monitor during waking hours and also log their diet for a minimum of 3 days either on paper or by using a Fitbit account created specifically for them. We found that in this population gathering diet and physical activity data using wearable technology is feasible and could potentially have clinically relevant effects on improving health outcomes. However, the results also demonstrated that, while monitoring the daily physical activity of pediatric patients was feasible, consistent dietary intake data collection had a lower compliance rate with the children in this study. Given this is a population that typically must monitor diet intake relatively closely, it does raise the question of whether this modality would be successful in children with other health conditions. 

The primary reason for those who did not reach clinical compliance was, indeed, due to a lack of dietary logs. Even though 84% of our study sample were compliant with wearing the Fitbit as instructed, only 63% recorded a complete diet for at least three days. Additional investigations regarding this aspect of the protocol may need more validation with research studies looking at reliable ways to collect such information. However, with such a large percentage of the subjects complying with wearing a physical activity monitor, this study would indicate that incorporating a wearable activity monitor as a vital sign in clinical practices to collect additional health information is not only feasible but also practical considering the benefit potential. 

A recent study from Coleman et al. examined the validity of an exercise vital sign for use in an outpatient electronic medical record and reported a high discriminate validity when incorporating self-reported measures of weekly moderate-to-vigorous physical activity into patient records during routine clinical visits [1]. However, when compared to other studies with similar demographics using more objective measures, such as accelerometers, the study authors acknowledged that the total minutes being reported by their sample may have been overestimating the total number of minutes spent in moderate-to-vigorous physical activity. If used with more reliable objective measures, this type of exercise vital sign would provide more information regarding physical activity behavior that could assist in the treatment of certain clinical populations, such as T1D, while also helping patients to better understand the impact daily physical activity can have on their body and diabetes management. Prior studies among general populations only investigated physical activity benefits and counseling outside a typical clinical setting, or accumulated amounts using self-reported measures [20,21]. Additionally, studies look at the benefits of physical activity broadly. Therefore, it is important to individualize physical activity counseling to the patient’s needs.

To our knowledge, this is the first study to explore the implementation of activity monitoring into standard clinical practice and test whether or not it would be feasible to use with pediatric patients. Prior studies have looked into the feasibility of a physical activity and/or exercise intervention to determine if children with T1D would adhere, but none have included measures that would also serve as a vital sign within medical records such as data from consumer-based physical activity monitors. Marrero and colleagues conducted a 12-week home-based intervention that included unsupervised moderate-to-vigorous physical activity routines using videos. Not only did they find this to be a safe and effective approach in which children would comply, but they also reported a reduction in HbA1c following the intervention [22]. In contrast, another investigation by Wong et al. found no change in HbA1c following 12 weeks of a home-based exercise intervention that also relied on video instruction and was unsupervised [23]. They attributed the lack of significant findings to a small sample size and possible low adherence since all the reported activity was completed in phone interviews in which researchers relied on young children to self-report how much moderate-to-vigorous physical activity they accumulated each week [23]. Only one study was identified that investigated the use of an accelerometer as a tool included as part of diabetes management. An investigation by Stenerson et al. tested an algorithm created from a simulation study by their group [24] that included data points from an accelerometer combined with a heart rate monitor as a form of threshold-based insulin pump suspension for insulin delivery. After identifying a potential accelerometer-augmented pump suspension algorithm, they further tested its effectiveness in reducing the incidence of exercise-related hypoglycemia in a small sample of 18 children playing soccer. They did not find significant differences in exercise-associated hypoglycemia compared to subjects on their usual basal rates, but acknowledged that a larger sample size may have achieved statistical significance [25]. Further, the algorithm was developed using a sample that was instructed to “go about their everyday activities” and not specific to exercise or large bouts of moderate-to-vigorous activity like what was completed in the effectiveness pilot study to test the same algorithm [24,25].

In a secondary analysis, we compared HbA1c for those who are compliant compared to non-compliant. Surprisingly, we found there to be a decreasing trend for those who were in full compliance with wearing the activity monitor and recording dietary intake. This was unexpected due to the small sample size but shows promise as future studies will look at the impact activity monitors may have in encouraging behavior change and provide additional framework within the recommendations for diabetes management. Doing so would help diabetes providers and staff to provide more informed guidance to their patients when it comes to managing T1D by having more reliable information regarding daily activity habits and intensity levels. It would also assist in the recommendations they would provide for carbohydrate intake and insulin adjustments prior to exercise or participation in recreational sports. The results of this study will allow researchers to further evaluate its possible integration into the health management of children with T1D. Future studies should look into the effectiveness of providing activity monitoring as part of routine medical care and a more holistic team approach when making adjustments to carbohydrate and insulin needs. 

When it comes to the potential out of pocket costs to patients, activity monitor price points have a wide variety of ranges depending on not just the specific brand, but also individual models they provide. Smart watches are beginning to explore incorporating glucose monitoring abilities, which may help overcome this obstacle, but if still required to pay out of pocket, some patients may need assistance financially. Some insurers are already utilizing accelerometers within their existing plans offered to employees, as well as strategies to deploy them, but, to our knowledge, most plans are not providing coverage for these devices as part of their plans. Research has also shown that people in general would be more willing to try consumer-based activity monitors if they were provided to them by their primary care doctor’s office, helping them to incorporate these devices into their daily lives [26].

Limitations to this study include the lack of a control group, the small sample size, and recruiting a very specific population that included only children with T1D. However, this study demonstrates that it would be feasible to monitor daily physical activity and diet using consumer-based devices instead of relying on patient self-reporting during appointments with their clinician. Other studies have established accelerometer data to be more accurate than self-report measurements of physical activity [3,27]. It is also important to note the added value this tool could offer if used by competitive athletes to improve glycemic control and performance while keeping them safe, which cannot be overstated enough. When used in conjunction with current diabetes management devices and adequate nutrition, ideal metabolic set points could be easier to achieve during sports and competitive activities, which would enhance overall performance while lessening the risk of severe hyper- and/or hypoglycemia. Future studies should investigate the possible incorporation of this technology into the health management of T1D. It is also important that this technology be explored in all clinical populations, including routine annual wellness visits with a primary care physician. More robust investigations are also needed for validating the use of wearable activity monitors beyond three days, since research suggests a minimum of four days with at least ten hours of wear time during waking hours for the reliable determination of weekly physical activity behaviors. This would be ideal for general populations as a preventative measure and for the management of co-morbidities for patients with chronic disease. 

## 5. Conclusions

This study highlights the feasibility and potential value of obtaining objective activity data that are more reliable and accurate as compared to information that is traditionally obtained by self-report or not at all. Consumer-based devices appear to be a feasible way to obtain physical activity and diet information from children with T1D. There is now overwhelming evidence of the known benefits of physical activity on cardiovascular health, mental health, and insulin sensitivity; therefore, physical activity should be considered a vital sign and be incorporated into patients’ medical record just like blood pressure and BMI [2,5]. Future studies need to work on the incorporation of this technology into diabetes care management for people of all ages and expand on these findings by looking into the health benefits of long-term changes in activity behaviors when incorporated in healthcare settings. By doing so, it is possible that diabetes management providers and staff may identify possible trends in glucose levels with real-world data providing feedback to also help make more informed decisions about changes in a patient’s care or treatment plan.

## Figures and Tables

**Table 1:** Demographic characteristics for all participants and separated by compliancy.

Characteristics	Enrolled *n* = 57	Compliant *n* = 36	Non-Compliant *n* = 21
Age, years	14 ± 3	13 ± 2.5	14 ± 3.5
HbA1c (%)	8.20 ± 1.30	8.19 ± 1.25	8.21 ± 1.43
Gender	Male: 36 Female 21	Male: 24 Female 12	Male: 12 Female 9
Race/Ethnicity	White: 49 (86%) Black: 5 (9%) Latino: 3 (5%)	White: 32 (88%) Black: 2 (6%) Latino: 2 (6%)	White: 17 (81%) Black: 3 (14%) Latino: 1 (5%)
BMI (Z-Score)	65.95 ± 23.41	65.89 ± 25.03	66.05 ± 18.47
T1D Diagnosis (Months)	52.77 ± 64.26	60.00 ± 73.48	40.86 ± 42.69
Insulin Pump Use	35 (61%)	21 (58%)	14 (67%)
Insurance Type	Private: 49 (84%) Medicare/Medicaid: 6 (12%) None: 2 (4%)	Private: 35 (97%) Medicare/Medicaid: 0 (0%) None: 1 (3%)	Private: 14 (67%) Medicare/Medicaid: 6 (28%) None: 1 (5%)
CGM Use	46 (81%)	27 (75%)	18 (86%)
Glucose (Avg)	185.46 ± 34.03	184.72 ± 32.95	188.20 ± 40.50
Daily Steps	10328.35 ± 3741.44	10752.83 ± 3338.98	9152.29 ± 4631.04 †
Vigorous Activity (mins.)	23.83 ± 22.78	24.34 ± 22.41	22.42 ± 23.88 †
Moderate Activity (mins.)	32.77 ± 20.49	34.74 ± 21.87	27.32 ± 17.74 †
Light Activity (mins.)	235.75 ± 68.51	253.20 ± 65.84	187.51 ± 50.86 †
Sedentary (mins.)	846.48 ± 244.53	794.29 ± 217.97	991.01 ± 267.18 †

†: *n* = 13 (number of participants compliant with activity monitor but not diet log). Abbreviations: HbA1C = glycated hemoglobin; T1D = Type 1 Diabetes; CGM = continuous glucose monitor; BMI = body mass index.

**Table 2:** Results of compliance.

Compliance Metric	Yes (%)	No (%)
Activity Monitor	48 (84%)	9 (16%)
Diet Record	36 (63%)	21 (37%)
Clinically Compliant	36 (63%)	21 (37%)

## Data Availability

The data presented in this study are available on request from the corresponding author.
